# Analysis of a Sardinian Multiplex Family with Autism Spectrum Disorder Points to Post-Synaptic Density Gene Variants and Identifies *CAPG* as a Functionally Relevant Candidate Gene

**DOI:** 10.3390/jcm8020212

**Published:** 2019-02-07

**Authors:** Elena Bacchelli, Eleonora Loi, Cinzia Cameli, Loredana Moi, Ana Florencia Vega Benedetti, Sylvain Blois, Antonio Fadda, Elena Bonora, Sandra Mattu, Roberta Fadda, Rita Chessa, Elena Maestrini, Giuseppe Doneddu, Patrizia Zavattari

**Affiliations:** 1 Department of Pharmacy and Biotechnology, University of Bologna, 40126 Bologna, Italy; cinzia.cameli@gmail.com (C.C.); elena.maestrini@unibo.it (E.M.); 2 Department of Biomedical Sciences, Unit of Biology and Genetics, University of Cagliari, 09042 Cagliari, Italy; e.loi14@studenti.unica.it (E.L.); lorymoi@gmail.com (L.M.); aflvegabe@gmail.com (A.F.V.B.); slvbls@gmail.com (S.B.); fadda85@gmail.com (A.F.); 3 Department of Medical and Surgical Sciences, DIMEC, St. Orsola-Malpighi Hospital, University of Bologna, 40138 Bologna, Italy; elena.bonora6@unibo.it; 4 Department of Biomedical Sciences, Unit of Oncology and Molecular Pathology, University of Cagliari, 09124 Cagliari, Italy; mattu.sandra@yahoo.it; 5 Department of Pedagogy, Psychology, Philosophy, University of Cagliari, 09123 Cagliari, Italy; robfadda@gmail.com; 6 Center for Pervasive Developmental Disorders, AO Brotzu, 09134 Cagliari, Italy; richess@libero.it (R.C.); iosettodoneddu@aob.it (G.D.)

**Keywords:** autism spectrum disorder, ASD, CAPG, VDAC3, postsynaptic density proteins, PSD proteins

## Abstract

Autism spectrum disorders (ASDs) are a group of neurodevelopmental disorders with high heritability, although their underlying genetic factors are still largely unknown. Here we present a comprehensive genetic characterization of two ASD siblings from Sardinia by genome-wide copy number variation analysis and whole exome sequencing (WES), to identify novel genetic alterations associated with this disorder. Single nucleotide polymorphism (SNP) array data revealed a rare microdeletion involving *CAPG*, *ELMOD3,* and *SH2D6* genes, in both siblings. *CAPG* encodes for a postsynaptic density (PSD) protein known to regulate spine morphogenesis and synaptic formation. The reduced *CAPG* mRNA and protein expression levels in ASD patients, in the presence of hemizygosity or a particular genetic and/or epigenetic background, highlighted the functional relevance of *CAPG* as a candidate gene for ASD. WES analysis led to the identification in both affected siblings of a rare frameshift mutation in *VDAC3*, a gene intolerant to loss of function mutation, encoding for a voltage-dependent anion channel localized on PSD. Moreover, four missense damaging variants were identified in genes intolerant to loss of function variation encoding for PSD proteins: *PLXNA2*, *KCTD16*, *ARHGAP21,* and *SLC4A1*. This study identifies *CAPG* and *VDAC3* as candidate genes and provides additional support for genes encoding PSD proteins in ASD susceptibility.

## 1. Introduction

Autism spectrum disorder (ASD) (Mendelian Inheritance in Man (MIM) 209850) is a neurodevelopmental disorder characterized by early onset in childhood, atypical and repetitive behaviors and impairments in communication and reciprocal social interaction. The phenotype of ASDs is extremely heterogeneous, with individual differences in a wide range of symptoms and severity [1,2,3,4].

Moreover, comorbidity with other neurological and behavioral disorders, such as developmental delay/intellectual disability, obsessive–compulsive disorder, attention deficit hyperactivity disorder, depression, anxiety disorder, social phobia, bipolar disorder, seizures, and others, is frequently observed.

The estimated prevalence of ASD in the worldwide population is 1 in 160 children and appears to be increasing [5], making it a major burden to society. Autism is more prevalent in male individuals, with an overall male-to-female ratio close to 3:1 [6].

Multiple lines of evidence support the strong role of genetics and heritability in the etiology of ASDs [2,3,4]. However, in line with the highly heterogeneous phenotypic manifestations, the underlying genetic architecture of ASD is also complex, with a combined contribution of both common and rare forms of genetic variations [7].

The major advances in the field of molecular diagnostic technology, such as the development of chromosome microarray technology (CMA) and whole-exome sequencing (WES), have allowed to identify more than 800 genes and loci, harboring rare de novo and inherited copy number variants (CNVs) and single nucleotide variants (SNVs), predisposing to ASDs. Even if the contribution of each gene/locus to ASD risk is very low, most of them converge on a smaller set of specific pathways such as synaptic function, transcriptional regulation and chromatin remodeling. In particular, alteration of synaptic homeostasis may be a common biological process associated with ASDs [8]. Indeed, many genes involved in synaptic function, and notably postsynaptic density (PSD) genes, a huge protein complex crucial for synaptic transmission and plasticity, were shown to play a role in ASD and other neurodevelopmental disorders [9].

Despite the major advances that have been made in recent years in our understanding of the genetic architecture of ASD, causative mutations remain unknown in the majority of ASD patients.

The Sardinian population has often proved to be fundamental for genetic studies of association in monogenic but also multifactorial diseases. The complex multi-locus and multi-allelic nature of certain diseases has indeed been unveiled studying Sardinian cohorts [10,11,12,13,14], other times showing founder effects only detectable in genetic isolates [15,16].

In the last years, CNV analysis by microarray technology has become the first-tiered genetic investigation for ASD, in particular when the affected individual shows also intellectual disability. However, CNV analysis can explain only a portion of ASD genetic architecture, given that the ASD phenotype may be the result of multiple variants of different size and effect, possibly affecting different genes involved in the same biological pathway. Therefore, even in presence of relevant CNVs, combining CNV analysis with sequencing data is warranted in order to better understand the role of different variants in the ASD phenotype [17].

Here we present a comprehensive genetic characterization of two ASD sibling from Sardinia. We identified a rare 2p11.2 deletion involving the whole coding region of *CAPG*, a member of postsynaptic density genes, and part of *ELMOD3* and *SH2D6* genes. We evaluated the impact of the deletion on CAPG expression. Finally, we identified deleterious variants in genes intolerant to loss of function mutations that could have a role in ASD susceptibility in combination with the 2p11.2 deletion.

## 2. Material and Methods

### 2.1. Participants

The Sardinian family participating in this study consists of father (AUT003.1), mother (AUT003.2), and two affected brothers (AUT003.3 and AUT003.4). Genomic DNA obtained from blood was available for all members of the pedigree.

Both siblings were diagnosed using the Autism Diagnostic Observation Schedule—Generic (ADOS) protocols [18] plus clinical evaluation, according to the VI Diagnostic and Statistical Manual of Mental Disorders (DSM-IV) criteria.

The older brother, named AUT003.3, received a diagnosis of Pervasive Developmental Disorder—Not otherwise Specified (PDD—NOS) when he was 7 years old, according to the diagnostic criteria of the DSM-IV. The results of the module II of the ADOS showed a deficit in the reciprocal social interaction with restricted and repetitive interests, difficulty in the changes of the routine of daily life, significant global motor clumsiness and deficit of eye contact. At that time, his non-verbal intellectual functioning was in the norm (Leiter-r Intelligence Quotient, IQ = 106).

The younger brother, named AUT003.4, received a diagnosis of Autism Disorder when he was 4 years old (DSM-IV). The results of the module I of the ADOS showed impairment in communication and language refers to a framework of autism, and in reciprocal social interaction, corresponding to a spectrum disorder. He also presented restricted and repetitive interests and stereotypies. At that time, his non-verbal intellectual functioning was in the norm (Leiter-r Intelligence Quotient, IQ = 87). He also showed a significant delay in receptive and expressive language, with aspects of immediate echolalia and poor spontaneous verbalization. At the age of 10, he showed a significant cognitive delay (IQ = 46). The ADOS at the age of 10 indicates difficulties in the areas of communication and reciprocal social interaction, as well as restricted and repetitive interests and stereotypies.

The Social Responsiveness Scale: Adult Research Version (SRS: ARV) and the Broad Autism Phenotype Questionnaire (BAPQ) were used in order to assess the parents’ Broader Autism Phenotype (BAP) (Table 1). Both parents did not exceed clinical cutoffs in BAP total measures (BAPQ Total and SRS: ARV Total). Only the father exceeded BAPQ score cutoffs for the “Social Behavior” (Aloof) subscale. In general, the father shows higher scores than the mother in all the measures of the BAPQ and the SRS: ARV. In addition, to exclude a possible diagnosis of ASD, parents were also assessed with the Autism Diagnostic Observation Schedule (ADOS—module IV), that resulted to be negative for both parents.

Simultaneously with the AUT003 family, blood samples were taken from two unaffected children of the same age of probands AUT003.3 and AUT003.4, to be used as experimental controls in the expression studies and in the sequencing of the *CAPG* promoter.

Subsequently, blood samples of 13 ASD probands and 22 healthy control subjects were collected to test the expression levels of *CAPG* mRNA. The 13 ASD probands were recruited at the Stella Maris Clinical Research Institute for Child and Adolescent Neuropsychiatry (Calambrone, Pisa, Italy). ASD diagnosis was based on the Autism Diagnostic Interview-Revised (ADI-R) and ADOS. A clinical evaluation was undertaken in order to exclude known syndromes associated with autism. Standard karyotyping, fragile-*X* testing, electroencephalogram (EEG), and array-based comparative genomic hybridization (aCGH) were obtained for all probands. The control sample consists of 22 unrelated Italian individuals with no psychiatric disorders.

This study was approved by the local Ethical Committee and took place in observation of the declaration of Helsinki.

### 2.2. DNA and RNA Samples Extraction

Genomic DNA was extracted from peripheral whole blood lymphocytes using salting-out procedure. DNA was quantified with NanoDrop (NanoDrop Products, Thermo Scientific, Wilmington, DE, USA) and by fluorometric reading (Quant-iT™ PicoGreen^®^ dsDNA Assay Kit, Thermo Fisher Scientific, Waltham, MA, USA).

Total RNA from all members of family AUT003, 13 ASD probands, and 24 healthy control subjects was extracted from PBMCs (Peripheral Blood Mononuclear Cells, isolated over a Ficoll-Hypaque density gradient) using Qiagen RNAeasy mini kit (Qiagen, Hilden, Germany) and quantified with NanoDrop. cDNA was synthesized using the High Capacity Kit (Applied Biosystems, Carlsbad, CA, USA). Then, 40 ng of cDNA were used to test *CAPG* and *ELMOD3* gene expression by quantitative reverse transcription PCR (qRT-PCR) using SYBR Green in all individuals.

### 2.3. CNV Analysis

Genotyping was performed using Human1M-Duo DNA Analysis BeadChip^®^ (Illumina, San Diego, CA, USA), which interrogate 1.2 million loci, according to the manufacturer’s instructions.

The clustering algorithm implemented in GenomeStudio (V2010.3) was used to cluster the data. GenomeStudio was also used to evaluate all genotypes using a quantitative genotype quality score called GenCall (GC) score, ranging from 0 to 1 with 1 being the best. The quality score cutoff was set at 0.15. Single nucleotide polymorphism (SNP) quality control (QC) was performed according to recommended guidelines [19].

Genome-wide CNVs were detected using a multiple-algorithm approach to maximize sensitivity and specificity of CNV calling. CNVs required calling by at least two different CNV detection algorithms out of these three: PennCNV [20], QuantiSNP [21], and CNVPartition (Illumina, San Diego, CA, USA). The required data for CNV analysis, (i.e., between-sample normalized fluorescence (Log R ratios (LRR) and B allele frequency (BAF) values) and genotypes for each sample), were exported directly from Illumina’s GenomeStudio software. To reduce false positive calls, we excluded CNVs if failed stringent quality-control criteria: spanning <10 probes, low confidence score (confidence score <15), more than 50% of their length overlapping segmental duplications and if they were within centromere proximal cytobands. As a final step, we joined CNVs that appeared to be artificially split by either of the calling algorithms.

The physical coordinates of each CNV, base pair (bp) position along the chromosome, were based on Build 37 (hg19) of the human genome assembly. CNVs passing QC were finally annotated to RefSeq genes, within 10 kb beyond the 5′- and 3′-untranslated regions (UTRs), to include CNVs extending to neighboring genes or overlapping potential regulatory regions. Similarly, we annotated CNVs overlapping exons.

We further filtered these stringent genic CNVs to identify a subset of “rare” CNVs, defined as CNV calls overlapping (>75%) copy number stable regions of the genome [22].

We manually inspected all rare CNVs by plotting their Log R ratios (LRR) and B allele frequencies (BAF).

### 2.4. CNV Characterisation and Segregation Analysis

In order to validate rare interesting CNVs in family AUT003, quantitative PCR experiments were performed using Fast SYBR Green (Thermo Fisher Scientific, Waltham, MA, USA). Each assay was conducted in triplicate, with at least three sets of primers corresponding to the region of interest and another mapping to a control region on *FOXP2* gene at 7q31.1. The number of copies of each amplified fragment was calculated using the ΔΔCt method. All family members (the parents and both siblings) were tested for confirming inheritance and segregation of CNVs.

To help resolve the microdeletion breakpoints, long range PCR across the 2p11.2 deletion was then carried out using the KAPA HiFi HotStart DNA Polymerase following the manufacturer’s suggested protocol (primers GGTGAAAATCTCCATTCCTTGA and CGACCCAGGCTTTAGTTATCAG).

The 241-bp deletion-spanning amplicon was purified and sequenced using Sanger method.

### 2.5. Gene Expression Analysis

*CAPG* and *ELMOD3* transcript levels were tested in family AUT003, in a commercially available cDNA panel (Human MTC Panel I, Clontech, Mountain View, CA, USA) including five different tissues (brain, testis, kidney, colon, prostate, thyroid) and in cDNA derived from blood of all member of family AUT003 and two unaffected controls by qRT-PCR with SYBR Green using 40 ng of cDNA. *CAPG* transcript levels were also assessed in 13 ASD probands and 22 additional healthy control subjects by qRT-PCR. To amplify *CAPG* fragment, we designed the forward primer in exon 4 and reverse primer in exon 5 (primers: CAGGGCAATGAGTCTGACCT and GGCTCCTGTGGAGGTCTTGT; 102 bp amplicon length; NM_001256140.1); for *ELMOD3* fragment, we designed forward primer in exon 5 and reverse primer in exon 6 (primers: GGTGAAAGATTGTCTGCTGGA and AGAATGCCATGGTTCTTCAA; 104 bp amplicon length; NM_001135022.1). All the data were normalized using the housekeeping gene *TFRC* as reference gene.

### 2.6. Western Blot

Proteins from lymphocytes were isolated using the Membrane Protein Extraction Kit (Mem-PERTM Plus, Thermo Fisher Scientific, Waltham, MA, USA) following the manufacturer’s instructions. Protein extracts were fractioned by 10% Sodium dodecyl sulfate (SDS) polyacrylamide gel and separated at 100 V during 90 min. Then, the proteins were transferred onto 0.45 µm nitrocellulose membrane, blocked in 5% milk overnight at 4 °C and then incubated 2 h with primary antibody anti-CAPG (1:1000, 10194-1-AP, Proteintech, Rosemont, IL, USA). After hybridization with primary antibody, the membrane was washed with Tris-buffered saline containing Tween-20 three times, then incubated with horseradish peroxidase-labeled secondary antibody (Jackson ImmunoResearch, Cambridge House, UK) for 1 h at room temperature and washed with Tris-buffered saline containing Tween-20 three times. Final detection was performed with Luminol-based Enhanced Chemiluminescent (ECL) Western blotting reagents (BIO-RAD, Hercules, CA, USA). An antibody against GAPDH (1:500, MAB374, Millipore, Burlington, MA, USA) was used for gel-loading control. Western blot signals were quantified using ImageJ program. The intensity of each band was normalized respect to that of GAPDH. The assay was performed in duplicate.

### 2.7. Whole Exome Analysis

DNA from the two affected siblings was captured using the Nextera exome enrichment kit (Illumina Inc., San Diego, CA, USA) according to the manufacturer’s protocol and sequenced as 100-bp paired-end reads on the Illumina HiSeq2500 platform (Illumina Inc., San Diego, CA, USA).

Exomes had a read depth (DP) of 10× or more for 90% of the total exome coverage and 20× or more for 80%. The raw data generated by WES were analyzed using CoVaCS, a recently developed pipeline for Next Generation Sequencing data analysis [23]. CoVaCS generates a final set of variants using a consensus call-set approach from three different algorithm (GATK, Varscan and Freebayes).

VCF (Variant Call Format) files of the CoVaCS consensus calls were annotated using Annovar [24]. The gene based annotation (position, nomenclature, gene name, gene function) was performed using RefSeq.

In order to remove low-quality variants called genotypes were required to have DP ≥10, and Genome Quality (GQ) ≥20. We selected only rare variants with MAF ≤1% in gnomeAD exome, gnomeAD genome [25] and the 1000 Genomes Project [26]. We considered in our analysis only variants located at exons or splicing sites; furthermore, we selected only Likely Gene Disrupting (LGD) and damaging missense mutations. LGD mutations included stop-gain, stop-loss, frameshift and splicing mutations.

Damaging missense mutations were defined as deleterious by at least two of the following criteria: SIFT (Sorting Intolerant From Tolerant) score ≤0.05, Polyphen2 (HDIV) score ≥0.95, Mutation Assessor ≥2, Phred transformed CADD (Combined Annotation-Dependent Depletion) score ≥15, placental mammal PhyloP ≥2.4, vertebrate PhyloP ≥4 [27].

Synaptic genes and PSD genes were defined as previously described [28,29].

## 3. Results

### 3.1. Identification and Characterisation of CAPG Deletion in Family AUT003

During a genome-wide CNV scan using a multi-algorithm approach on one multiplex family with ASD from Sardinia, we identified a rare genic heterozygous deletion of ~37 kb, involving the whole *CAPG* gene, the last coding exons of *ELMOD3* and the first non-coding exon of *SH2D6*.

**Figure 1:**
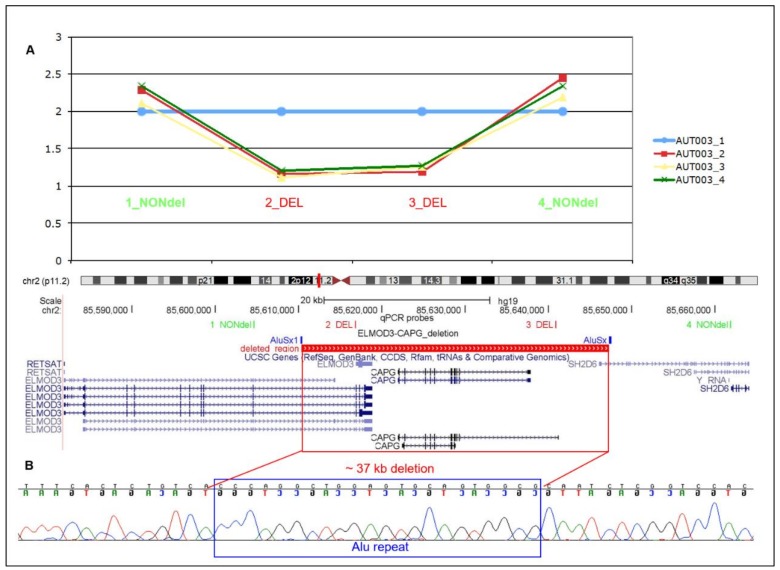
Validation and breakpoint definition of chr2p11.2 (*ELMOD3-CAPG-SH2D6*) deletion in family AUT003. (**A**) The microdeletion was confirmed in the unaffected mother and the two affected siblings by quantitative PCR using four different probes. The genomic position of the deletion is indicated with a red rectangle; (**B**) Sequence electropherogram: the 21-bp breakpoint-spanning sequence, common to both ends, is pinpointed by a blue rectangle (NCBI build 37 coordinates). DEL: assay located in the deletion; NONdel: assay located outside the deletion.

We validated the microdeletion, confirming that it was inherited from the unaffected mother to both the affected siblings (Figure 1A). Analysis of chromosome 2 SNPs (mapping in a region of 138 kb including the deletion) indicates that the non-deleted paternal copy of *CAPG* was also shared identical-by-descent in all two affected children.

To determine whether this rare CNV may have been mediated by repetitive elements, we mapped the breakpoints at base pair resolution. At the deletion junction we identified the presence of 21 bp mapping both in chr2:85,610,292-85,610,313 and in chr2:85,647,268-85,647,289 inside two Alu repeats with high homology (AluSx1, chr2:85610243-85610455 and AluSx, chr2:85647216-85647540) (NCBI, National Center for Biotechnology Information, build 37; Figure 1B), suggesting that this deletion is likely to be generated through non-allelic homologous recombination. We resolved the size of the deletion of 36,977 bp.

CNV analysis of this family allowed us also to identify two additional rare exonic CNVs: a chr3q26.32 deletion including exon 1 of *KCNMB2*, transmitted from the unaffected mother to affected son AUT003.3, and a chr6q21 duplication involving the first four exons of *BEND3* and the last four exons of *PDSS2,* inherited from the unaffected father by affected son AUT003.3 (Supplementary Figure S1).

### 3.2. ELMOD3-CAPG-SH2D6 Deletion Frequency in ASD Cases and Controls

In order to assess the relevance and test the frequency of *ELMOD3*-*CAPG*-*SH2D6* in ASD susceptibility in autism and control populations, we exploited large-cohort whole-genome CNV data already available. We identified two rare deletions encompassing *ELMOD3*-*CAPG*-*SH2D6* in two simplex ASD families from the AGP genome-wide study including CNV data of 2147 European ASD families [30], and in both cases the deletion was inherited from the father (Sample_ID: 5424_3 and 5536_3) [30]. Recently, a homozygous deletion affecting the last three exons of *ELMOD3*, the entire *CAPG* gene, and the first non-coding exon of *SH2D6* has been identified in a Tunisian ASD proband with ID and hearing impairment. The deletion is inherited from consanguineous parents, both heterozygous for the deletion [31]. No CNVs in this genomic region are present in 6639 European controls [32,33,34,35,36]. Indeed, this genomic region is considered a copy number stable region, according to the CNV map of the human genome [22].

To exclude the hypothesis that the identified deletion might be relatively common in the Sardinian population, we analyzed 80 Sardinian control samples finding that no control sample showed the deletion.

### 3.3. CAPG and ELMOD3 Gene Expression Analysis

**Figure 2:**
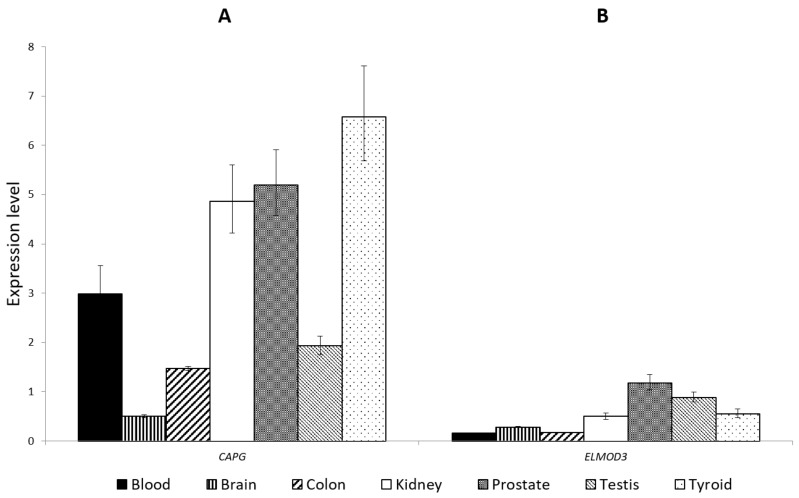
*CAPG* and *ELMOD3* gene expression levels in different tissues. (**A**) *CAPG* expression levels; (**B**) *ELMOD3* gene expression levels Bar plots represent fold change with upper/lower limits relative to *TFRC* reference gene.

In order to obtain a global overview of the *CAPG* and *ELMOD3* expression, we evaluated their expression levels in different human tissues and two unaffected controls. We observed the presence of *CAPG* and *ELMOD3* transcripts in all tested tissues, including brain (Figure 2).

**Figure 3:**
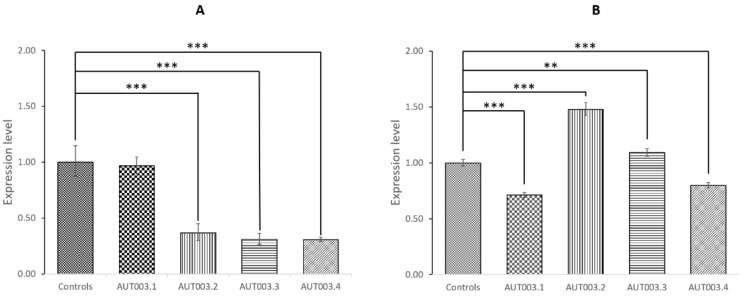
*CAPG* and *ELMOD3* gene expression levels in AUT003 family members and in controls. (**A**) *CAPG* expression levels; (**B**) *ELMOD3* gene expression levels. Bar plots represent fold change with upper/lower limits relative to controls. The statistical comparisons were conducted between AUT003 family members and the average expression level between the two controls. Asterisks indicate statistically significant differences (** *p* <0.01, *** *p* <0.001).

We did not test *SH2D6* expression since the deletion involves only *SH2D6* non-coding exon 1. Moreover, from the GTExPortal gene expression data, *SH2D6* is reported to be expressed at very low levels in brain and its expression is barely detectable in whole blood. Then we tested *CAPG* and *ELMOD3* expression levels in all members of the AUT003 family and in two healthy controls. *CAPG* expression was significantly reduced (*p* <0.001) in the mother and in the two ASD siblings carrying the heterozygous deletion, compared to two healthy controls who do not carry the deletion (Figure 3A). Conversely, we did not observe a reduction of *ELMOD3* levels in the two affected sibling and their mother (Figure 3B) but the expression levels were highly variable between the tested individuals.

**Figure 4:**
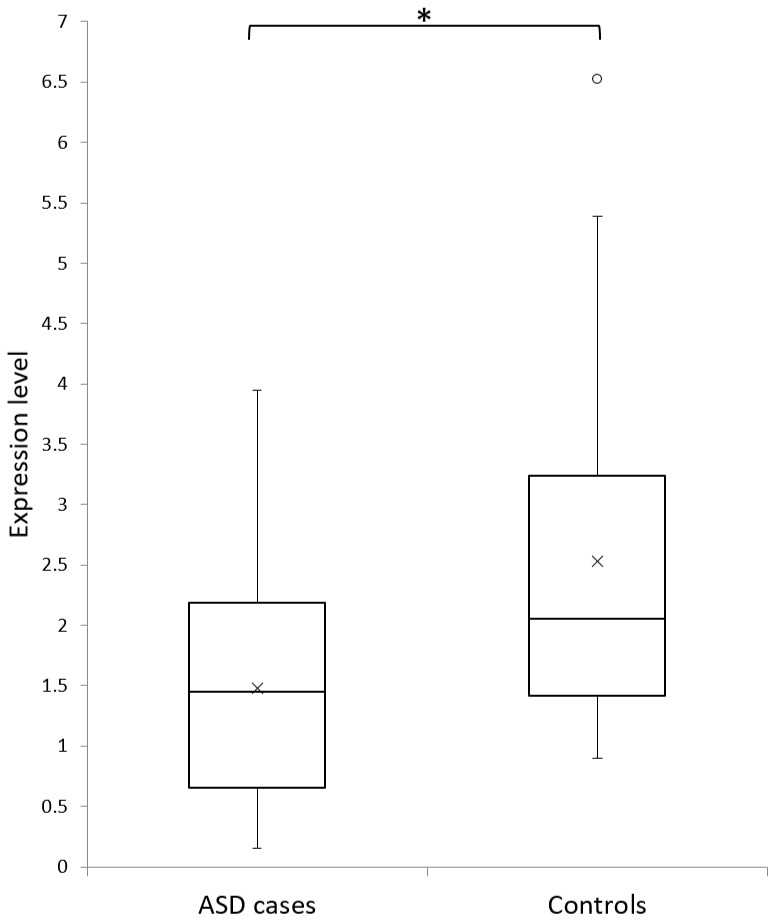
*CAPG* gene expression levels in autism spectrum disorder (ASD) cases and in controls. Boxplot of the relative *CAPG* expression of 13 ASD cases and of 22 healthy controls. The expression values were normalized using *TFRC* reference gene. Mean values are represented by crosses. The asterisk indicates a statistically significant difference (*p* = 0.026).

We also performed a quantitative characterization of *CAPG* expression in an independent cohort of 13 Italian ASD probands and 22 unaffected individuals. We observed a significant reduction of *CAPG* expression in ASD probands compared with healthy control subjects (Mann–Whitney test *p* = 0.02647) (Figure 4). Since all ASD probands were between 4 and 18 years of age while in our control samples there were also 12 older subjects (age range: 21–47 years), we repeated the analysis only considering the 10 controls aged 8 months–12 years. The difference between ASD and control children was still significant (Mann–Whitney test *p* = 0.03493), excluding a possible age effect influencing *CAPG* expression.

### 3.4. CAPG Protein Expression Analysis

**Figure 5:**
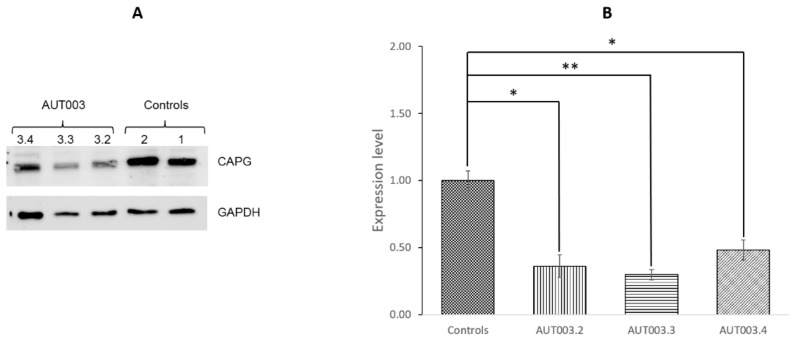
CAPG protein expression levels in AUT003 family members and controls. (**A**) Representative blots of CAPG protein expression in autism spectrum disorder (ASD) cases and controls. GAPDH was used as loading control; (**B**) CAPG expression. Bars represent mean ± SD (standard deviation). The statistical comparisons were made between AUT003 family members and the average between the two controls. Asterisks indicate statistically significant differences (* *p* <0.05, ** *p* <0.01).

The expression protein level of CAPG was determined in the two siblings affected by ASD, their unaffected mother and in two healthy control children. The Western blot analysis showed significant lower CAPG expression levels in the AUT003 family members with the deletion compared to healthy control subjects (Figure 5).

### 3.5. Whole Exome Sequencing

We performed a WES analysis for AUT003.3 and AUT003.4 by Illumina Nextera technology.

The 81.3–87.5% and 94.6–98.7% of target regions showed at least 20× and 10× coverage for AUT003.3 and AUT003.4, respectively.

In the first step of WES analysis, we focused on LGD variants given their known deleterious effect on protein function (Table 2). In particular, we restricted the analysis on variants in genes intolerant to loss-of-function (LoF) variants based on the pLi score, i.e. probability of being loss-of-function (LoF) intolerant (pLi) [37]. Among the 33 identified LGD variants, three are in genes strongly intolerant to loss of function variants (pLi score ≥0.9). Two of these variants, located in *SIK1* and *VDAC3*, are shared by both affected siblings. The variant in *SIK1* disrupts a splice site and it is inherited from the unaffected mother, while the variant in *VDAC3* is a frameshift deletion transmitted by the unaffected father. The effect of the *VDAC3* 2 bp-frameshift deletion is the introduction of five novel amino acids followed by premature termination codon probably inducing nonsense-mediated decay. According to GTExPortal gene expression data, SIK1 is expressed at low levels in brain, while VDAC3 is highly expressed in brain and, interestingly, it encodes for a postsynaptic density member, CAPG.

To comprehensively inspect the load of potentially damaging variant shared by both affected siblings, we also investigated the presence of damaging missense variants in genes with a pLi score ≥0.9. Among the 20 shared variants identified in genes intolerant to mutation, four affect PSD genes: *PLXNA2*, *KCTD16*, *ARHGAP21*, and *SLC4A1* (Table 3).

No damaging variants were identified in the hemizygous *CAPG* genomic region, thereby excluding a complete *CAPG* knock-out in both affected siblings.

## 4. Discussion

Genome-wide CNV analysis in a Sardinian multiplex family led to the identification of a rare genic heterozygous deletion on chromosome 2, involving the whole *CAPG* gene, the last coding exons of *ELMOD3* and the first non-coding exon of *SH2D6*, inherited by both affected siblings from the unaffected mother. The deletion encompasses a CNV stable region of genome [22] and has not been reported in controls, while similar deletions spanning *CAPG*-*ELMOD3*-*SH2D6* have been detected in three independent ASD families, in the heterozygous [30] and the homozygous [31] state, suggesting that this deletion might be involved in ASD susceptibility.

Interestingly, *CAPG* expression analysis in AUT003 family showed a significant reduction of mRNA (*p* <0.001) and protein expression (*p* <0.05), due to hemizygosity, in all carriers of the identified deletion (mother and both affected siblings). Moreover, we observed a significant reduction of *CAPG* mRNA expression levels in 13 ASD Italian probands compared to 22 unaffected controls (*p* = 0.026), suggesting that decreased CAPG levels might represent a risk factor with a low effect size for ASD. We could speculate that differences in CAPG gene expression could likely be due to the presence of different genetic and/or epigenetic background in ASD subjects compared to controls.

CAPG, capping actin protein gelsolin like, is an excellent candidate for neurologic development abnormalities, given that it is essential for dendritic spine development and synapse formation [38]. Abnormality in spine number and shape has been observed in a number of neurological disorders, including autism, and contributes to brain dysfunction [39]. Recently, it has been showed that CAPG is present in dendritic spines of cultured hippocampal neurons and the branched actin filament network containing CAPG appears to be a prominent feature of the spine head [40]. Capg knockdown in rat hippocampal neurons cultures resulted in a marked decline in spine density accompanied by increased filopodia-like protrusions and consequently a reduced number of functional synapses [38]. Moreover, through a computational approach to improve the reconstruction of the PSD network it has been suggested that CAPG interacts with SRC at the postsynaptic density [41]. Overall, these findings indicate that capping of actin filaments by CAPG represents an essential step for the remodeling of the actin architecture underlying spine morphogenesis and synaptic formation during development.

Interestingly, CAPG has also been implicated in Rett syndrome, a genetic neurodevelopmental disorder caused by variants in MECP2. In particular, transcriptomic data analysis obtained from human cells with impaired MECP2 function suggests that MECP2 regulates CAPG expression and that CAPG could be involved in altered cytoskeleton organization observed in Rett syndrome [42]. Moreover, Mecp2-null mice showed Capg upregulation that might be involved in the molecular mechanisms leading to altered neuronal architecture observed in Rett syndrome [43].

Given the presence of *CAPG* deletion also in the unaffected mother, we can hypothesize that only a recessive loss of function is sufficient to cross the risk threshold to develop ASD, while the presence of hemizygous *CAPG*, or even its reduced levels of expression, could confer a smaller increase in susceptibility and lead to the ASD only in association with other genetic risk variants.

Therefore, a WES analysis has been performed for both affected siblings, in order to search for additional genetic alterations that could act in combination with *CAPG* deletion to determine ASD phenotype. Among all rare variants identified by WES analysis, we focused first on LGD variants given their harmful effect on protein function. Moreover, since it has been reported that genes have a different tolerance for deleterious variants, we considered only LGD variants in genes reported to be intolerant to loss-of-function (LoF) variants based on pLi score [37]. The most interesting variant is a frameshift deletion in *VDAC3* gene, shared by the two siblings and inherited from the unaffected father. The *VDAC3* 2 bp-frameshift deletion determines the introduction of five novel amino acids followed by premature termination codon but also should lead to mRNA degradation owing to nonsense-mediated mRNA decay. *VDAC3* encodes the voltage-dependent anion channel 3 and it is a member of human postsynaptic density (hPSD) genes highly expressed in several brain regions including the hypothalamus and caudate nucleus. VDACs are a family of proteins present on the mitochondrial outer membrane, on which they supply diffusion pores for small hydrophilic molecules and are involved in the release of cytochrome c at the onset of apoptotic cell death. Interestingly, recent evidence suggests that VDAC could be involved in the pathogenesis of several syndromes, including autism [44,45,46]. In particular, Gonzalez-Gronow and colleagues identified the presence of autoantibodies against VDAC proteins in autistic individuals, suggesting their possible causal role in the neurologic pathogenesis of autism [44]. Moreover, Crane and colleagues have hypothesized that the beneficial effect recorded in patients with autism following treatment with coenzyme Q, or other agents influencing the transport of electrons, may be due to the control of such molecules on the porin channels [45]. Therefore, the *VDAC3* frameshift mutation identified in this study might contribute to the ASD phenotype in the two siblings.

Recent studies suggested that the autism phenotype may be influenced by the combined effects of variants in multiple genes involved in common pathways. Thus, it is worthy of note that the two affected siblings share disrupting mutations (a microdeletion and a frameshift mutation) in two different genes, both encoding for proteins associated to the PSD, a huge synaptic protein complex linked to various neuropsychiatric diseases including neurodevelopmental disorders such as ASD [29].

Further evidence for a possible involvement of PSD genes in modulating the ASD phenotype in this family is provided by the identification of damaging missense mutations shared by the two affected siblings in four additional LoF intolerant genes (pLi score ≥0.9) encoding for PSD proteins: *PLXNA2*, *KCTD16*, *ARHGAP21,* and *SLC4A1*.

Of particular interest is the damaging mutation involving *PLXNA2*, a gene specifically expressed in fetal and adult brain [47]. This gene encodes the plexin A2, a member of the plexin-A family of semaphorin co-receptors, a large family of secreted or membrane-bound proteins that mediate repulsive effects on axon pathfinding during nervous system development. Interestingly, reduced PLXNA4 expression, another plexin-A family member, have been observed in individual with ASD [48], while a genetic variant in *PLXNA2* has been associated with schizophrenia [49]. Moreover, it has been suggested that PlxnA2 is an inhibitor of the GTPase Rap1B-regulating granule cell (GC) progenitors in the hippocampal neuroepithelium in a Rap1-dependent manner [50]. Interestingly, similar to PlxnA2 GAP activity, Shank3, one of the stronger ASD candidate genes, inhibits Rap1 GTPases. In particular, two different SHANK3 mutations associated to ASD led to Rap1 activity reduction [51]. In addition, Plxna2−/− mice showed deficits in associative learning, sociability, and sensorimotor gating in behavioral studies [50]. Altogether, this evidence suggests that variants in *PLXNA2* contribute to neuropsychiatric disorders.

The other three shared variants in synaptic genes were identified in *KCTD16*, *ARHGAP21*, and *SLC4A1*. *KCTD16* encodes for an auxiliary subunit of GABA-B receptors that modulate both pre and postsynaptic GABA-B receptor response [52,53,54,55]. Interestingly, a *KCTD16* polymorphism has been associated with epilepsy by a genetic linkage study [56].

*SLC4A1* encodes for a member of the ion transporter SLC4 family, that mediate the pH regulation and transepithelial ion transport, critical for several biological processes, including modulation of neuronal excitability [57,58,59,60]. Dysfunctions of SLC4 transporters are associated with a broad range of diseases, including central nervous system disorders, such as intellectual disability, migraine, epilepsy, autism and drug addiction [61,62,63,64,65,66].

Finally, *ARHGAP21* is a widely expressed gene with high levels in brain and muscle [67]. It encodes for a Rho GTPase activating-protein, localized to the Golgi complex through binding to GTP-bound ARF1. Arhgap21 protein acts as a Rho-GAP for Cdc42 and regulates the Arp2/3 complex and actin cytoskeleton remodeling, playing a role in Golgi complex organization [68]. Interestingly, CAPG plays a central role in the assembly of the actin-related protein 2/3 (Arp2/3)-generated branched actin networks, by capping the actin filament barbed end, which promotes the Arp2/3-dependent filament nucleation and optimal branching [69,70,71].

Therefore, both ARHGAP21 and CAPG have a crucial role in actin assembly control, through the Arp2/3 complex regulation.

In conclusion, this study identified *CAPG* as an excellent candidate gene in ASD susceptibility and allowed the identification of new promising candidate genes encoding for PSD proteins, thus contributing to the comprehensive framework for understanding this complex and heterogeneous disorder. Indeed, we can hypothesize that the injurious effect of inherited *CAPG* deletions can be modified by the specific genetic background of the carrier individual. Therefore, in the Tunisian ASD proband [31], the presence of *CAPG* homozygous deletion could be sufficient to determine the autistic phenotype, while, in the two ASD Sardinian siblings here described, the *CAPG* deletion might act in combination with the *VDAC3* 2 bp-frameshift deletion and/or with the other identified deleterious variants in the PSD genes (*PLXNA2*, *KCTD16*, *ARHGAP21,* and *SLC4A1*). However, further studies are warranted to better clarify the role of CAPG, alone and in combination with the other identified PSD genes, in the impairment of synaptic homeostasis that is considered a common biological process associated with ASDs.

## Figures and Tables

**Table 1:** Assessment of parents’ autism phenotype using the Broad Autism Phenotype Questionnaire, the Social Responsiveness Scale, and the Autism Diagnostic Observation Schedule.

Measure	Mother	Father
**BAPQ Scores**		
Aloof	2.4	3.5
Pragmatic Language	2.2	2.7
Rigidity	2.2	2.7
BAPQ Total	2.4	3
**SRS: ARV scores**		
Awareness	10	8
Mannerism	8	10
Communication	9	21
Cognition	4	10
Motivation	3	10
SRS:ARV Total	34	59
**ADOS—IV Module Scores**		
Communication	0	0
Reciprocal Social Interaction	0	0

BAPQ: Broad Autism Phenotype Questionnaire; SRS: Social Responsiveness Scale; ARV: Adult Research Version; ADOS: Autism Diagnostic Observation Schedule–Generic. BAPQ, SRS: ARV, and ADOS–IV module scores are shown.

**Table 2:** LGD variants.

Identified Variant			pLi Score	Synaptic Genes [28,29]	Genotype	
Gene Base Change	AminoAcid Change	Gene			AUT003.3	AUT003.4
chr1:g.19559158delA NM_001271428:c.1739delT	p.F580Sfs*10	*EMC1*			0/1	0/0
chr1:g.24779922delT NM_001322854:c.565delT	p.L189Cfs*15	*NIPAL3*			0/1	0/0
chr1:g.108771659G>A NM_001143989:c.C1543T	p.R515X	*NBPF4*			0/1	0/1
chr1:g.158299688A>T NM_001764:c.T561A	p.Y187X	*CD1B*			0/1	0/1
chr2:g.30961265C>T NM_144575:c.1594+1G>A		*CAPN13*			0/1	0/0
chr2:g.58386928-58386929insTAAT NM_001114636:c.1114_1115insATTA	p.T372Nfs*12	*FANCL*			0/1	0/1
chr2:g.120439282C>T NM_001105198:c.C853T	p.R285X	*TMEM177*			0/1	0/1
chr3:g.38770224G>A NM_001293306:c.C2449T	p.R817X	*SCN10A*			0/0	0/1
chr3:g.188327410T>A NM_001167671:c.T891A	p.Y297X	*LPP*			0/0	0/1
chr4:g.8235241G>A NM_001318480:c.3054+1G>A		*SH3TC1*			0/1	0/1
chr6:43306235-43306238delTCTT NM_014345:c.5498_5501del	p.K1833Rfs*6	*ZNF318*			0/0	0/1
chr7:g.150028118C>T NM_138434:c.C625T	p.Q209X	*ZBED6CL*			0/1	0/0
chr7:g.150864372G>T NM_001098834:c.C264A	p.C88X	*GBX1*			0/0	0/1
chr8:g.42259530-42259531delAT NM_001135694:c.551_552del	p.H184Rfs*5	*VDAC3*	0.97	PSD; Excitability	0/1	0/1
chr8:g.145140986delC NM_003801:c.1824delC	p.C610Afs*43	*GPAA1*			0/0	0/1
chr10:g.102240866-102240872del NM_003393:c.353_359del	p.G120Wfs*37	*WNT8B*			0/1	0/0
chr10:g.123845143-123845144delCC NM_001291876:c.3128_3129del	p.P1044Tfs*4	*TACC2*			0/0	0/1
chr11:g.59577372dupC NM_017840:c.76dupG	p.A26Gfs*14	*MRPL16*			0/1	0/0
chr11:g.116701354G>A NM_000040:c.55+1G>A		*APOC3*			0/0	0/1
chr12:g.25705804C>T NM_001145728:c.89+1G>A		*LMNTD1*			0/0	0/1
chr12:g.54757526delC NM_020370:c.110delG	p.G37Afs*22	*GPR84*			0/1	0/0
chr12:g.64436690C>T NM_001346201:c.C610T	R204X	*SRGAP1*	0.99		0/1	0/0
chr12:g.133198246dupA NM_012226:c.889dupA	p.N298Kfs*6	*P2RX2*			0/0	0/1
chr15:g.75641682T>C NM_024608:c.434+2T>C		*NEIL1*			0/0	0/1
chr16:g.107113-107114insTC NM_024571:c.369_370insTC	p.F125Pfs*5	*SNRNP25*			0/1	0/1
chr18:g.71822640delT NM_014177:c.462+2T>-		*TIMM21*			0/0	0/1
chr18:g.77917844dupG NM_032510:c.940dupC	p.Q314Pfs*72	*PARD6G*			0/0	0/1
chr19:g.10204081dupA NM_001321411:c.1165dupT	p.S389Ffs*4	*ANGPTL6*			0/0	0/1
chr19:g.43859812G>C NM_020406:c.380-1G>C		*CD177*			0/1	0/1
chr20:g.61591931delT NM_022082:c.473delT	p.I158Tfs*11	*SLC17A9*			0/0	0/1
chr21:g.44839738C>A NM_001320643:c.1119+1G>T		*SIK1*	0.99		0/1	0/1
chr22:g.25124143C>T NM_001255975:c.1905+1G>A		*PIWIL3*			0/0	0/1
chrX:g.23019507dupT NM_182699:c.1334dupT	p.I446Nfs*35	*DDX53*			1/1	1/1

LGD: Likely Gene Disrupting; PSD: postsynaptic density; pLi: probability of being loss-of-function (LoF) intolerant (pLi).

**Table 3:** Rare damaging missense variants, shared by both affected siblings, in genes with pLi score ≥0.9.

Identified Variant			pLi Score	Synaptic Genes [28,29]	Genotype	
Gene Base Change	AminoAcid Change	Gene			AUT003.3	AUT003.4
chr1:g.109801543A>G NM_001408:c.A3800G	p.H1267R	*CELSR2*	0.9999992		0/1	0/1
chr1:g.117146504G>A NM_001007237:c.C1366T	p.R456C	*IGSF3*	0.987084919		0/1	0/1
chr1:g.162725022G>A NM_006182:c.G494A	p.R165Q	*DDR2*	0.990992372		0/1	0/1
chr1:g.208390903G>T NM_025179:c.C365A	p.S122Y	*PLXNA2*	0.993981182	PSD	0/1	0/1
chr2:g.96956078C>T NM_014014:c.G2728A	p.V910I	*SNRNP200*	1		0/1	0/1
chr2:g.239975199C>T NM_006037:c.G3172A	p.A1058T	*HDAC4*	0.999989727		0/1	0/1
chr4:g.126238072T>C NM_001291285:c.T506C	p.V169A	*FAT4*	0.999999998		0/1	0/1
chr5:g.141052986C>T NM_022481:c.G954A	p.M318I	*ARAP3*	0.967505029		0/1	0/1
chr5:g.143587022G>A NM_020768:c.G745A	p.A249T	*KCTD16*	0.959972608	PSD; Excitability	0/1	0/1
chr7:g.100491451G>C NM_000665:c.C403G	p.P135A	*ACHE*	0.996674942		0/1	0/1
chr9:g.88938095T>A NM_001185074:c.A2201T	p.E734V	*ZCCHC6*	0.999999563		0/1	0/1
chr10:g.70726833C>A NM_001256910:c.C760A	p.H254N	*DDX21*	0.998039216		0/1	0/1
chr10:g.79581043G>A NM_004747:c.C3199T	p.R1067C	*DLG5*	0.999998542		0/1	0/1
chr10:g.79590510C>G NM_004747:c.G1870C	p.E624Q	*DLG5*	0.999998542		0/1	0/1
chr10:g.24874455G>T NM_020824:c.C4763A	p.S1588Y	*ARHGAP21*	0.999946673	PSD	0/1	0/1
chr11:g.94918634T>G NM_144665:c.A548C	p.N183T	*SESN3*	0.994296083		0/1	0/1
chr11:g.114027109G>A NM_001018011:c.G1319A	p.R440Q	*ZBTB16*	0.980087306		0/1	0/1
chr12:g.112699118C>T NM_001109662:c.G2432A	p.R811H	*HECTD4*	1		0/1	0/1
chr17:g.42335485C>T NM_000342:c.G1151A	p.R384H	*SLC4A1*	0.909484295	PSD; Excitability	0/1	0/1
chr19:g.50100192G>A NM_020719:c.G2600A	p.R867H	*PRR12*	0.999969577		0/1	0/1

PSD: postsynaptic density; pLi: probability of being loss-of-function (LoF) intolerant (pLi).

## Supplementary Materials

The following are available online at http://www.mdpi.com/2077-0383/8/2/212/s1, Figure S1: Validation of chr3q26.32 (*KCNMB2*) deletion and chr6q21 (*BEND3*-*PDSS2*) duplication in family AUT003. (**A**) The genomic position of *KCNMB2* deletion, chr3:178246465-178511664 (NCBI build 37 coordinates), is highlighted in blue. The microdeletion was confirmed in the mother and the affected son AUT003_3 by qPCR using four different probes. (**B**) The genomic position of *BEND3*-*PDSS2* duplication, chr6:107408585-107546262 (NCBI build 37 coordinates), is highlighted in blue. The microduplication was confirmed in the father and the affected son AUT003_3 by qPCR using four different probes.
