# Supplementary figures and images for: Analysis of a Sardinian Multiplex Family with Autism Spectrum Disorder Points to Post-Synaptic Density Gene Variants and Identifies CAPG as a Functionally Relevant Candidate Gene
Source: J Clin Med. 2019 Feb 7;8(2):212. doi: 10.3390/jcm8020212 (PMC6406497; doi:10.3390/jcm8020212)

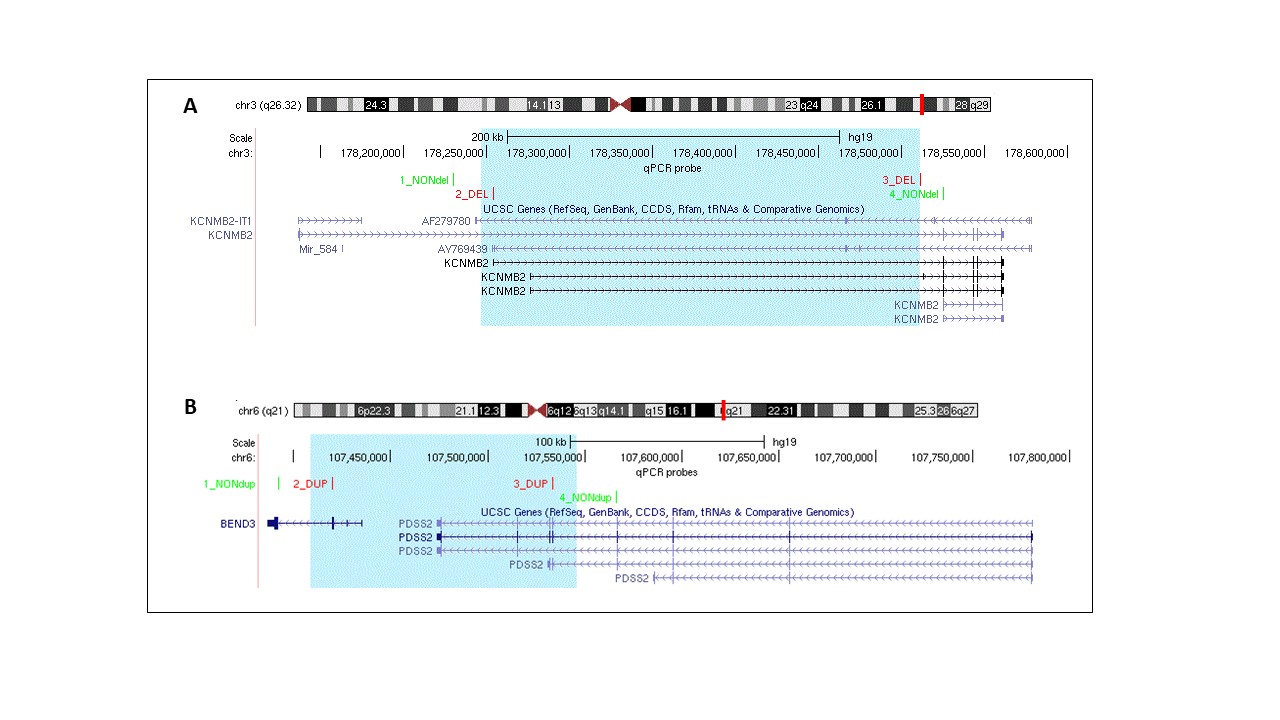

Supplement: Supplementary file 1 [file jcm-08-00212-s001.zip › jcm-437225-SI.jpg]
